# Synthesis, characterization, and imaging of radiopaque bismuth beads for image-guided transarterial embolization

**DOI:** 10.1038/s41598-020-79900-z

**Published:** 2021-01-12

**Authors:** Ayele H. Negussie, Quirina M. B. de Ruiter, Hugh Britton, Danielle R. Donahue, Quentin Boffi, Young-Seung Kim, William F. Pritchard, Chrit Moonen, Gert Storm, Andrew L. Lewis, Bradford J. Wood

**Affiliations:** 1 Center for Interventional Oncology, Radiology and Imaging Sciences, Clinical Center, National Institutes of Health, 9000 Rockville Pike, Bethesda, MD USA; 2 Biocompatibles UK Ltd, (a BTG, Now Boston Scientific Company), Lakeview, Riverside Way, Watchmoor Park, Camberley, GU15 3YL Surrey UK; 3 Mouse Imaging Facility, National Institute of Neurological Disorders and Stroke, National Institutes of Health, Bethesda, MD USA; 4 Radiation Oncology Branch, National Cancer Institute, National Institutes of Health, Bethesda, MD USA; 5 Center for Imaging Sciences, Imaging Division, University Medical Center Utrecht, Utrecht, 3584 CX The Netherlands; 6 Department of Biomaterials Science and Technology, University of Twente, Enschede, The Netherlands; 7 Department of Pharmaceutics, Utrecht University, Utrecht, The Netherlands; 8 Department of Surgery, Yong Loo Lin School of Medicine, National University of Singapore, Singapore, Singapore; 9 Center for Interventional Oncology, Radiology and Imaging Sciences, Clinical Center, Center for Cancer Research, National Cancer Institute, National Institutes of Health, Bethesda, MD USA

**Keywords:** Cancer, Chemistry, Materials science

## Abstract

Current therapy for hypervascular cancers, e.g., hepatocellular carcinoma, includes occlusion of the tumor blood supply by arterial infusion of embolic microspheres (beads) suspended in iodine-based contrast under fluoroscopic guidance. Available radiopaque, imageable beads use iodine as the radiopacifier and cannot be differentiated from contrast. This study aimed to synthesize and characterize imageable beads using bismuth as the radiopacifier that could be distinguished from iodine contrast based upon the difference in the binding energy of k-shell electrons (k-edge). Radiodense bismuth beads were successfully synthesized some with uniform bismuth distribution across the beads. The beads were spherical and could be infused through clinical microcatheters. The bismuth beads could be imaged with clinical dual-energy computed tomography (CT), where iodine-based contrast could be distinguished from the microspheres. The ability to separate iodine from bismuth may enhance the diagnostic information acquired on follow-up CT, identifying the distribution of the embolic beads separately from the contrast. Furthermore, with sequential use of iodine- and bismuth-based beads, the two radiopaque beads could be spatially distinguished on imaging, which may enable the development of dual drug delivery and dual tracking.

## Introduction

Embolic microspheres (beads) are used to occlude the vascular supply of tumors and may be loaded with chemotherapy drugs, which then elute from the microspheres and diffuse into the tissue. These drug-eluting beads are used for locoregional therapy, particularly for treating patients with unresectable hepatocellular carcinoma^1^. Embolic beads are typically made of inert hydrophilic materials with elastic properties and spherical geometry. These beads are generally non-radiopaque but can be rendered radiopaque by incorporation of a radioabsorber, such as iodine, zinc, tantalum, bismuth, or barium^2–6^. The development and use of imageable beads can inform direct real-time feedback during the embolization procedure, or on follow-up imaging^7,8^.

Recently, we have developed a radiopaque embolic bead through covalent attachment of iodinated groups onto the backbone of polyvinyl alcohol-*co*-acrylamido-2-methylpropane sulfonate precursor microspheres^2,9,10^. The radiopacity of the beads during delivery or follow up can facilitate identification, localization, and distribution of undertreated tumor^11,12^. It may also enable estimation of doxorubicin dose and spatial distribution in the liver^13^. One limitation of iodinated beads, however, is the challenge in distinguishing them from iodinated contrast agents using imaging based on X-ray absorption, i.e., fluoroscopy, cone beam CT, or CT imaging. During embolization, embolic agents (both radiopaque and non-radiopaque beads) are mixed with contrast agents to improve suspension and handling, and to estimate dosimetry and delivery of the beads to the tumor. Contrast may be somewhat retained, although intravascular contrast washes out over time^14^. Similarly, a contrast-enhanced CT scan after bead delivery may not normally differentiate among intravascular liquid contrast, iodinated beads, and contrast in stained parenchyma.

Attaching a radiopaque element with a higher atomic number, Z, and a different k-edge from iodine, may enable material decomposition and differentiation of a novel bead from iodine in contrast or from iodinated radiopaque beads using dual-energy CT (DECT)^15^. In DECT, two CT scans are simultaneously acquired at different energies, a low-energy image, e.g., 80 keV, and a higher-energy image, e.g., 140–150 keV, that can be processed to generate additional datasets that enable material decomposition. Bismuth has a k-edge of 90.5 keV compared to 33.2 keV for iodine and thus has the potential to be differentiated from iodine using DECT.

The purpose of this study was to synthesize and characterize novel imageable beads using bismuth as the radiopacifier. A secondary goal was to assess the ability to spatially differentiate the bismuth beads from iodine-based contrast agents using DECT imaging, as a requisite step towards dual tracked drug vectors in embolization therapies.

## Results

### Synthesis

**Figure 1:**
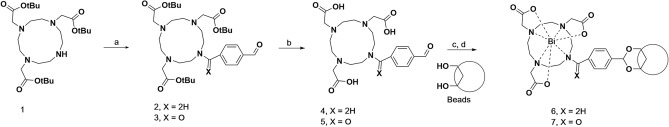
Synthesis of bismuth beads with an aromatic linker between the bead and the macrocycle. Reagent and condition: (**a**) 4-(bromomethyl)benzaldehyde, K_2_CO_3_/DMF or 4-formyl benzoyl chloride, DCM/Et_3_N, (**b**) HCl/dioxane, (**c**) MeSO_3_ in DMF or DMSO, 50 °C, (**d**) Bi(CF_3_SO_3_)_3_, pH 8–9 using NaHCO_3_, 80 °C or NMP/pyridine, 55 °C.

Reactive bismuth chelating agents were successfully synthesized by alkylation or acylation of the macrocycle **1** with bifunctional linkers, as shown in Fig. 1. These bifunctional linkers consisted of an aromatic ring with bromine or acid chloride on one end while aldehyde functional on the other end. Bead acetalation with the macrocycle was performed, and bismuth was then chelated onto the acetalated beads using Bi(CF_3_SO_3_)_3_ to give radiopaque beads **6** and **7** (Fig. 1).

The acetal formation between DO3A-4-formyl benzyl (**4**) and the sulfonated acylamido polyvinyl alcohol beads in DMSO solvent was a second-order reaction as the plot of the inverse concentration of (**4**) (M) versus time (min) showed a linear relationship with reaction rate constant, k = 8.5 × 10^−5^ L/mol min (Supplementary Fig. 1A). Similarly, acetal formation between DO3A-4-formyl benzyl (**4**) and the sulfonated acylamido polyvinyl alcohol beads in DMF solvent is also second order, but the reaction rate constant was tenfold faster, k = 8.5 × 10^−4^ L/mol min (Supplementary Fig. 1B).

### Characterization

Optical microscopy, elemental composition, scanning electron microscopy (SEM), and energy-dispersive X-ray analysis (EDX) were performed for both beads **6** and **7** to evaluate surface smoothness, the composition and uniformity of bismuth within and between beads.

#### Physical properties and elemental composition

The elemental chemistry of a dry bead is 27.28% C, 4.16% H, 2.58% N, 3.3% S, 35.23% Bi and 27.45% O and Na (combined) for bead **6** and 27.85% C, 3.87% H, 2.00% N, 3.18% S, 30.88% Bi and 32.22% O and Na (combined) for bead **7**. The percent bismuth content from the elemental composition analysis indicated a slightly higher bismuth content for bead **6** (35.23%) compared to bead **7** (30.88%). Other physical characteristics of different sizes of bismuth beads are shown in Supplementary Table 1.

#### Optical microscopic evaluation

**Figure 2:**
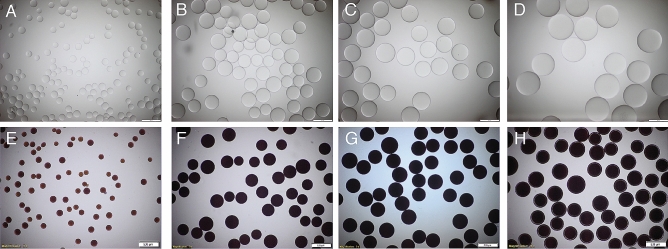
Light microscopy of beads. Size and appearance of different size of blank beads (**A**–**D**) and bismuth-functionalized beads (**7**) (**E–H**) under light microscopy, (**A**, **E**) 100–160 μm, (**B**, **F**) 250–355 μm, (**C**, **G**) 355–425 μm and (**D**, **H**) 425–600 μm.

The synthesized bismuth beads appeared dark brown and opaque under optical microscopy and maintained their spherical morphology and smoothness. However, the beads had a gross white appearance to the naked eye. The shapes and optical appearances for beads **7** are depicted in Fig. 2.

#### Scanning electron microscopy and energy dispersive X-ray analysis

**Figure 3:**
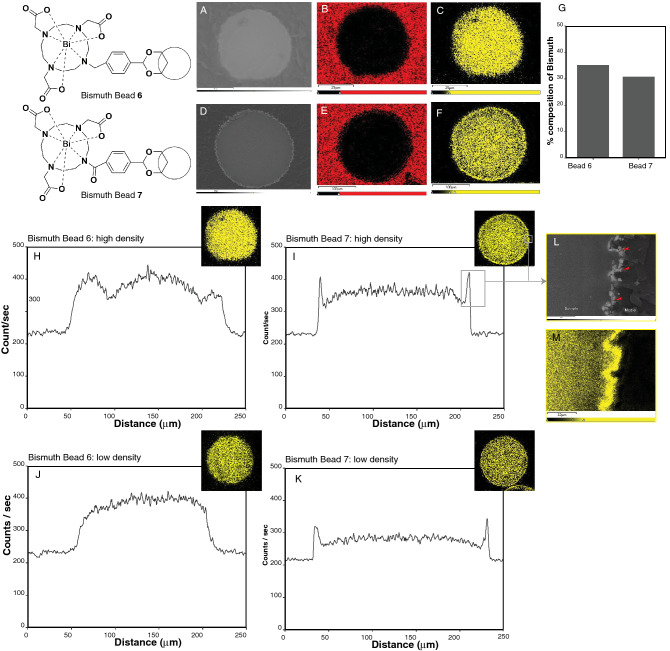
Scanning electron microscopy (SEM) and associated sulfur and bismuth elemental maps of cross-sections of bead **6 **and bead **7 **showing bismuth distribution. SEM image for bead **6** (**A**) and bead **7** (**D**). Associated Sulfur elemental maps for bead **6** (**B**) and bead **7** (**E**) and bismuth elemental maps for bead **6** (**C**) and bead **7** (**F**). (**G**) Bar plot of the average percent composition of bismuth in beads** 6** and **7**, showing a higher average bismuth concentration for bead **6** compared to bead **7**. Representative bismuth line scans of single beads with a high and low amount of bismuth, with the correlative bismuth elemental map in the upper right corner for each graph for bead **6** (**H**, **J**) and **7 (I**, **K**). For bead **6**, the maximum bismuth count/sec was relatively constant within each bead, reflecting a homogenous distribution of bismuth throughout the beads. For bead **7**, there was a peak in the bismuth counts/sec at the edge of the beads with otherwise homogeneous distribution of bismuth in the bead interior. Magnified views of the SEM (**L**) and bismuth element map (**M**) of the higher density bead **7** (**I**) showed an irregular bead edge and an accumulation of bismuth grains at the edge consistent with the bismuth peaks in the line graphs at both edges. Note that a slight increase in signal strength is present in the line scans between 20 and 200 μm. This effect is expected as the electron beam travels farther from the specimen edge. The effect is accentuated due to the small diameter of the spheres and may not indicate an actual increase in bismuth.

SEM revealed the sectioned internal structure of both beads **6** and **7** to be free of any visible pores (Fig. 3). Bead **6** appeared smoother than bead **7** which had an irregular margin and grain-like appearance at the bead periphery (Fig. 3). EDX elemental map and line scans revealed higher levels and more uniformly distributed bismuth across the sectioned bead **6,** compared to bead **7** (Fig. 3). Similar to the SEM results, bead **7** demonstrated irregularity at the periphery caused by a higher density of bismuth, as can be observed in the edge intensity in the line scan as well as in the EDX elemental map (Fig. 3). In addition, beads with higher and lower amounts of bismuth were identified. Figure 3H–K shows a line scan of both a high and a low bismuth containing bead for both bead **6** and bead **7**.

#### Microcatheter deliverability

Beads **7** (100–160 µm) were deliverable through 2.0 Fr and 2.4 Fr catheters, while larger beads (250–355 µm) required a minimum of a 2.4 Fr catheter to enable delivery without clogging (Supplementary Table 1).

### In vitro evaluation with MicroCT and clinical DECT

Beads **6** had higher bismuth content, and bismuth was homogenously spread from edge to edge than beads **7**. Therefore, bead **6** was selected for visualization with microCT and DECT.

#### MicroCT

**Figure 4:**
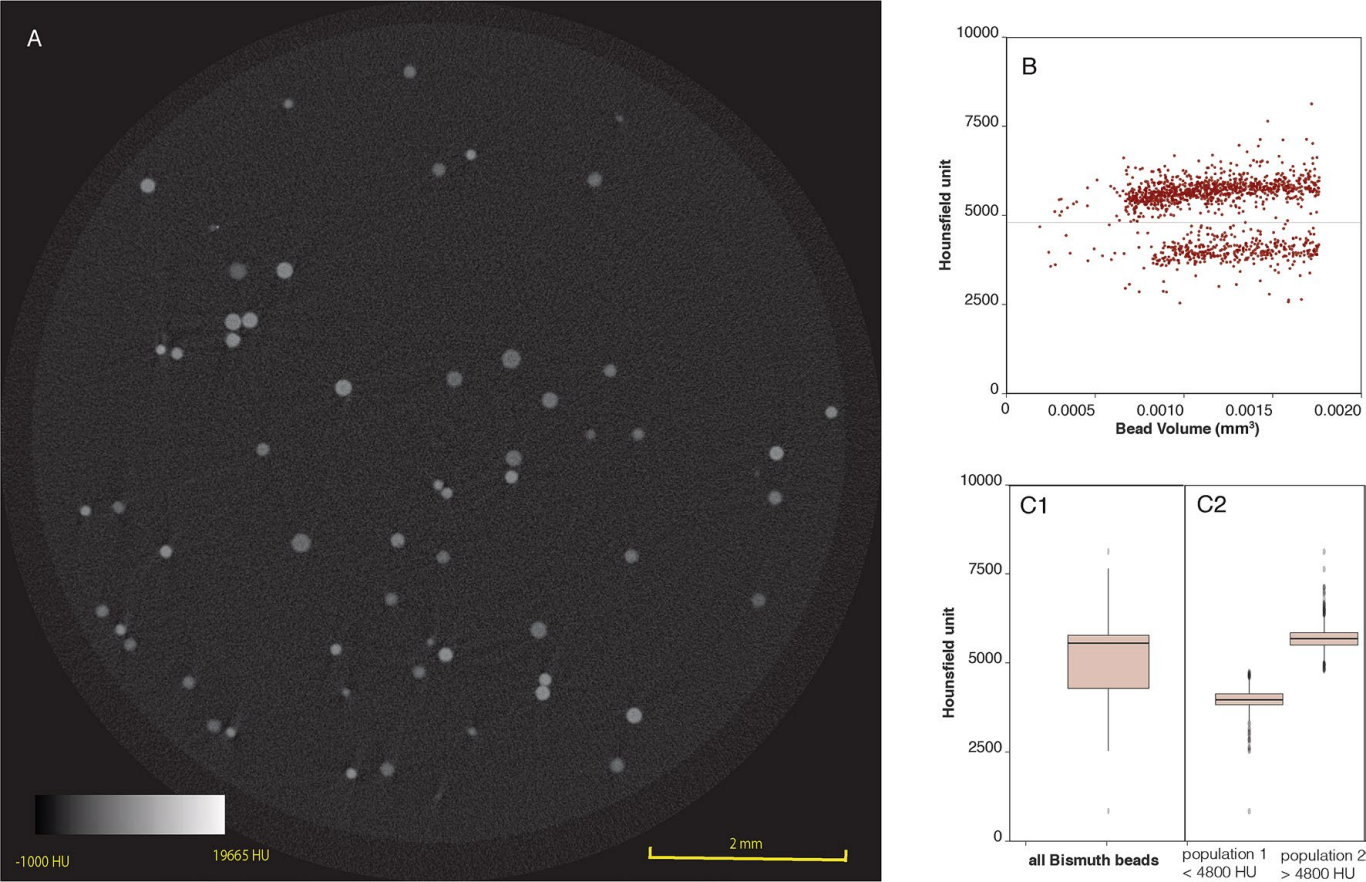
MicroCT imaging of bead **6**. (**A**) Axial slice of a suspension of bead **6**. (**B**) Scatterplot revealing two bead subpopulations that can be distinguished at a 4800 threshold. There was no correlation between bead volume and HU. (**C**) Boxplots of HU for all bismuth beads ranging 70–150 μm (**C1**) and both bead populations (**C2**) revealing the average intensity for both bead populations.

The radiopacity of bismuth bead **6** is presented in Fig. 4. A total of 1442 bismuth beads with a size range between 70 and 150 μm were selected for post-analysis after exclusion of beads near saturation on the image density scale, representing 1.4% of the original number. The mean Hounsfield units (HU) for the entire population of beads was 5200 ± 846 HU. There was no correlation between the bead volume and HU (Fig. 4); however, a histogram of the beads revealed two distinct HU intensity peaks. Dividing the beads into two groups with a 4800 HU threshold revealed that the lower density bead population had a mean HU of 3972 ± 365 (71% of the bead), while the higher density bead population had a mean HU of 5700 ± 325 (29% of the beads).

#### Dual energy CT

**Figure 5:**
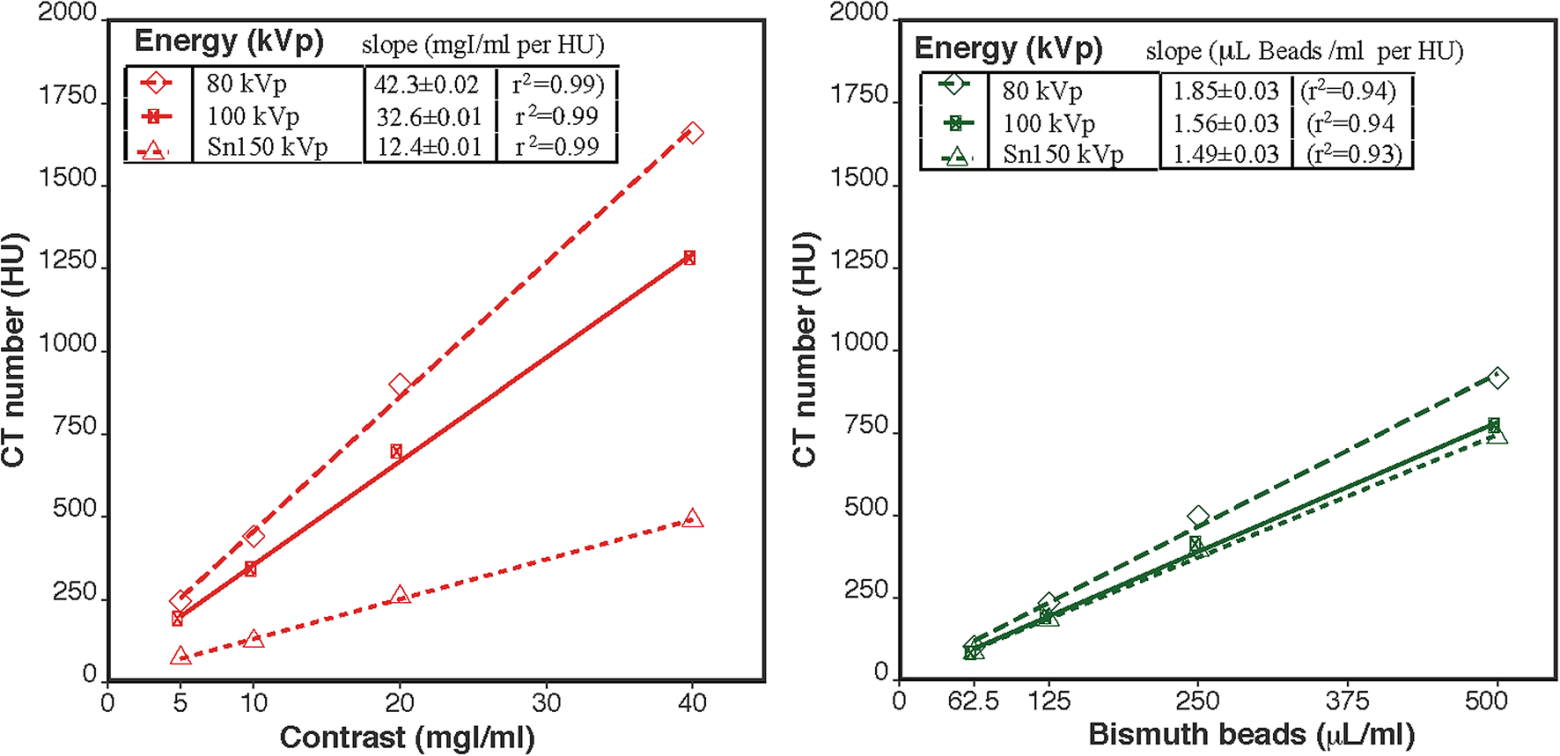
Clinical dual energy CT imaging. Linear regression between the image intensity or CT number (HU) for each of three energy levels and agarose gel content, either iodine (left) or bismuth beads **6** (right).

There was a linear correlation between CT number and both iodine concentration and bismuth bead volume/mL at each energy level (Fig. 5) of microsphere **6**. For iodine, the largest difference in HU value was observed between 80 kVp and Sn150 kVp acquisitions, as compared to the 100 and Sn150 kVp. For bismuth, the HU difference was slightly more between 100 and Sn150 kVp; however, both acquisitions would be feasible to image bismuth with minimum HU differences.

**Figure 6:**
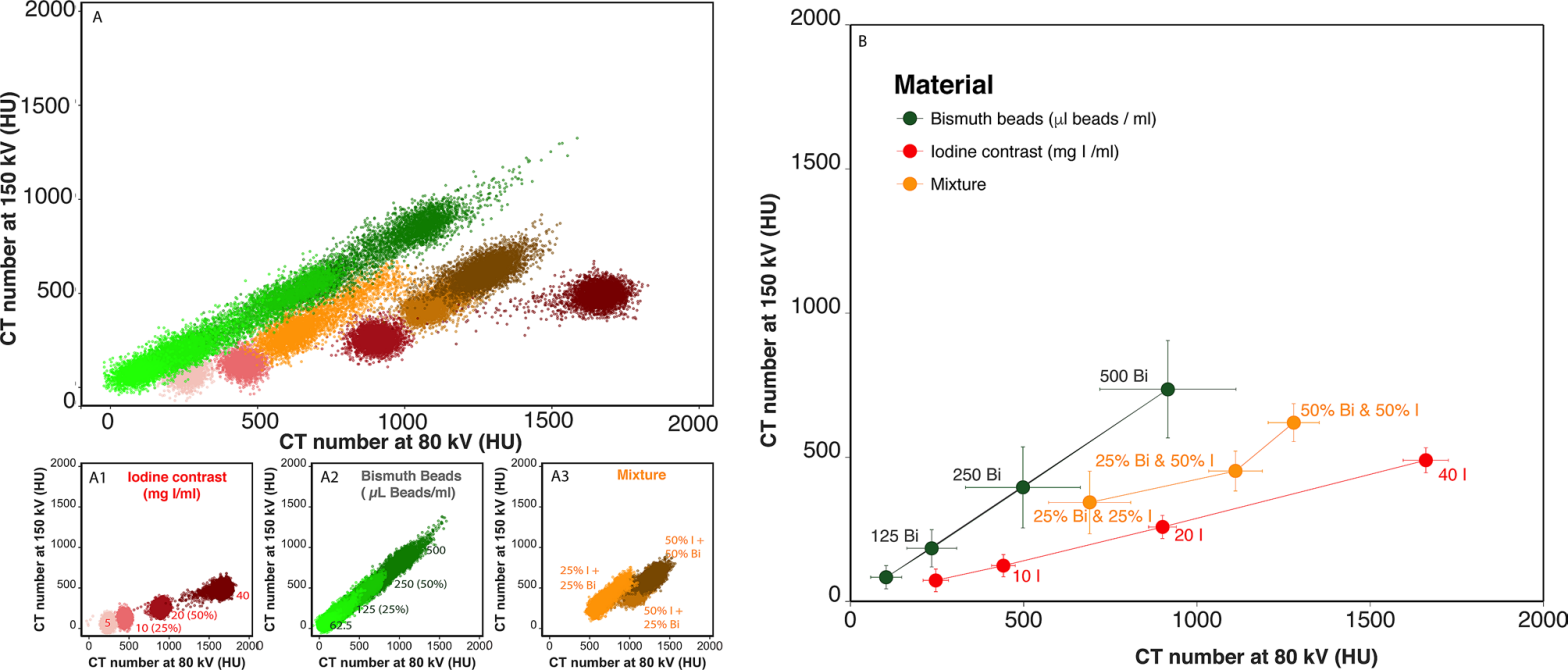
Correlation of CT numbers (HU) on dual energy CT at 80 kVp vs Sn150 kVp: iodine, bismuth beads (**6**) and mixture of both. (**A**) Scatter plots of the CT numbers (HU) derived from the 80 kVp and Sn150 kVp scans for each voxel. Subplots are shown as (**A1**) iodine (red), (**A2**) bismuth (green), and (**A3**) a mixture of both (orange). (**B**) The average and standard deviation (bars) of the CT number (HU) derived from the 80 kVp and Sn150 kVp scans for each tube.

**Figure 7:**
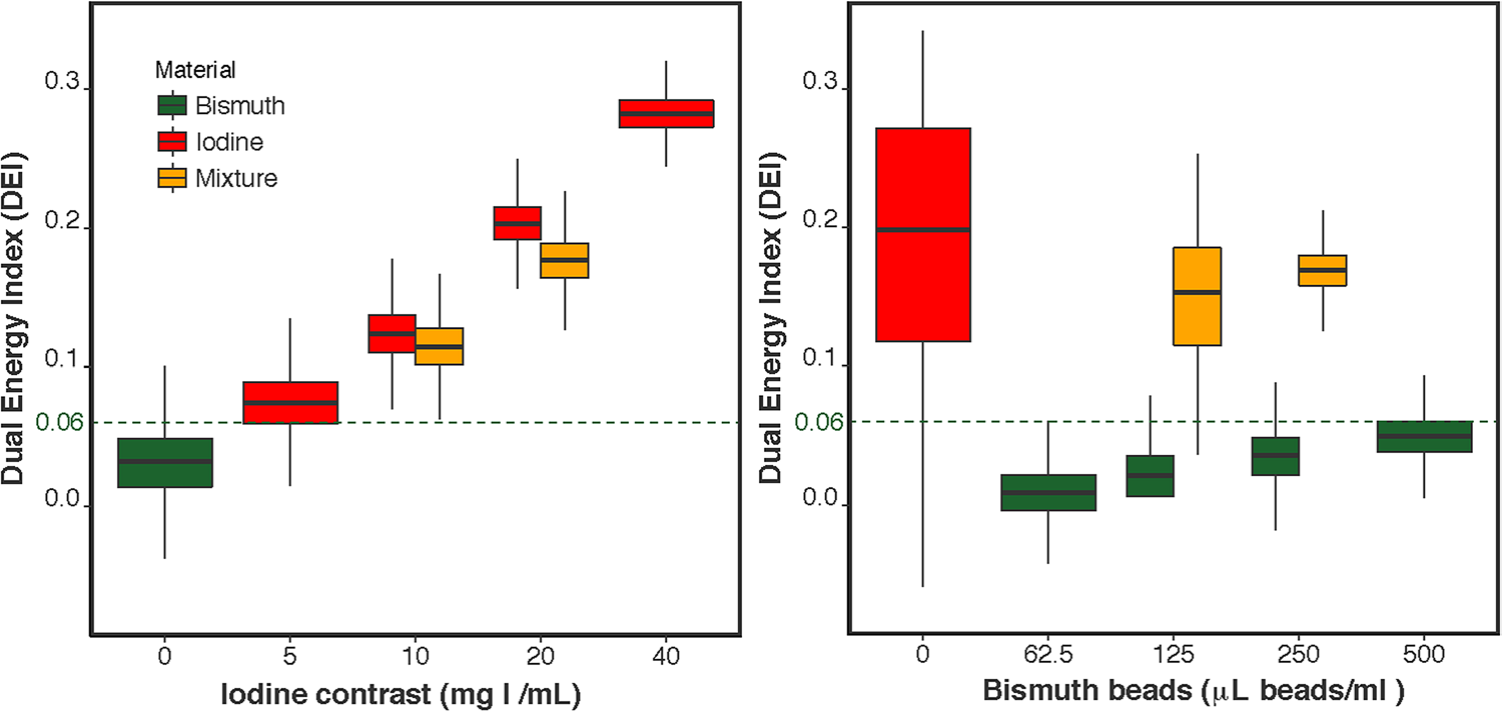
Boxplot of the dual energy index (DEI) calculated per voxel averaged per iodine concentration (left) or bismuth beads (right). The green line represents the DEI threshold of 0.06 which optimally classified iodine.

The final material decomposition analysis was performed using the 80 kVp and Sn150 kVp paired acquisition, as the iodine extraction was favorable for this acquisition pair. There was a good linear correlation between the iodine concentration and dual energy index (DEI) (r^2^ = 0.9, p < 0.01), while there was a poor linear correlation between the bismuth volume and DEI (r^2^ = 0.34, p < 0.01) as shown in Fig. 6. The DEI threshold that optimally separated iodine and bismuth was defined at 0.06. 99.9% of the voxels in the 10 mg I/mL tubes were above this threshold, while 99.7% of the 62.5 mL/mL voxels in the bismuth agarose solution were excluded (Fig. 7). For the mixed tubes, the HU of all voxels were above the threshold (Supplementary Table 2).

**Figure 8:**
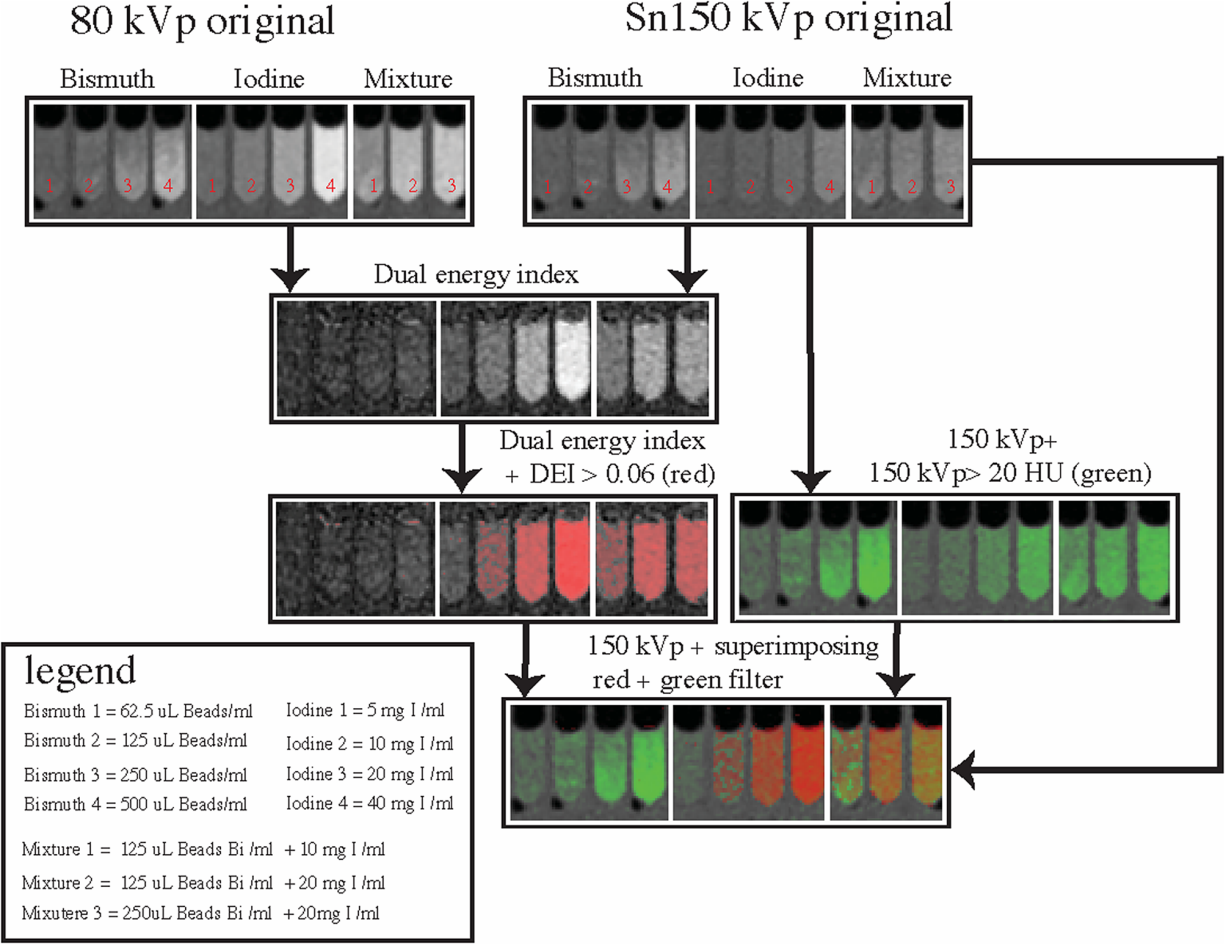
Dual energy CT post-processing workflow and outcome for bismuth, iodine and mixture agarose phantoms. Row 1: Original 80 kVp and Sn150 kVp energy reconstructions. Row 2: A dual energy index (DEI) image is calculated from the two scans. Row 3: DEI values greater than 0.06 are superimposed on the original DEI image as a red color map. (left image). Image intensity values above 20 HU in the Sn150 kVp image are superimposed on the original image as a green color map (right image). Row 4: Combined DEI and HU color maps as blended color maps superimposed on the Sn150 kVp image. High HU above the threshold (green) occurs for both iodine and bismuth, however, the increase in DEI (red) only occurs for iodine. Therefore, bismuth alone can be differentiated from either iodine alone or a bismuth/iodine mixture, due to the absence of red.

The DEI data derived from the 80 kVp and Sn150 kVp images and subjected to the 0.06 DEI threshold confirms that the imaging identifies tubes that contain iodine, whether alone or in combination with bismuth beads (Fig. 8). The HU map with the 20 HU threshold to exclude the agarose identified both iodine and bismuth within the tubes. The combination of the two maps (DEI map to identify iodine and HU to identify any radioabsorber) demonstrates that bismuth alone could be distinguished from iodine alone or the combination of iodine and bismuth.

## Discussion

In this study, two types of bismuth beads were synthesized having very similar linkers but with two different bismuth chelation techniques. For bismuth bead **6**, chelation was performed in aqueous media at pH 8–9, and for bismuth bead **7** an organic solvent was chosen. It was shown with elemental composition and SEM–EDX experiments that bead** 6** had a higher bismuth content and a more uniform distribution across the entire bead surface compared to bead **7**. Therefore, bead **6** was chosen for subsequent characterization and perhaps for future in vivo works. MicroCT of bead **6** showed that there were two populations of bead **6** with higher and lower radiographic density. Even though these two bead populations had different bismuth content, bismuth was relatively homogenous across the beads on SEM–EDX analysis. Both bead **6** subpopulations had a sufficiently high HU per bead to be clinically useful, with means of 3972 HU and 5700 HU. A plausible explanation for having two sets of beads with different bismuth contents might be related to variations in the number of macrocycles acetalated to the various beads, the amount of bismuth chelated to the macrocycle containing bead, or both. The overall radiopacity and standard deviation of the bismuth beads is in the same range as the radiopacity of the clinically utilized iodine-based radiopaque beads 4718 ± 257 HU^2^.

For clinical delivery, beads should have a stable suspension in liquid contrast and must be able to pass through a microcatheter^10^. The bismuth beads in the range of 100–160 μm were deliverable through both 2.0 and 2.4 Fr clinical microcatheters, while the larger beads (250–355 μm) required a 2.4 Fr microcatheter. Both beads sizes are comparable to those in standard clinical use.

DECT imaging may be used for material classification by exploiting the differences in X-ray absorption of iodine and bismuth from high radiographic density. This model could also be imposed in the clinical setting of liver tumor embolization. A DEI threshold of 0.06 distinguished iodine from the background and from bismuth beads alone. However, there was an overlap in DEI values for the higher concentrations of bismuth beads and the lower concentrations of iodine. Therefore, the DEI as a single image feature could be used to identify if iodine was present, either alone or in combination with bismuth beads. The model dictates that densities (HU) above background must be either iodine or bismuth, while any tube with a DEI below the threshold that does not contain iodine and must be bismuth beads. For embolization, the only density would be iodine or bismuth beads. Refining and optimizing the synthesis process to increase the selection of only the bead populations with higher radiographic density could lead to an increase in the signal to noise ratio in CT scanning and material decomposition. The DEI could be used to define and follow the washout of iodine contrast after embolization. Post-embolization residual tumor enhancement may be hard to distinguish from high attenuation iodine beads, without a subtraction image in conventional CT. Bismuth beads would not present this problem^16^. These novel bismuth beads have the potential to improve image-guided local drug delivery in patients with liver cancer by directly identifying either non-targeted delivery or undertreated tumor. The ability to choose from two bead formulations to load drugs opens the door to dual drug delivery and “dual drug dose tracking”. Two beads could be loaded with doxorubicin and a second agent, such as an immunomodulator. Two drugs could be selected and targeted with a mechanistic rationale that factors in the tumor microenvironment or molecular data. For example, doxorubicin intercalates in the nucleus and is more active for cell-cycle-specific regions of high mitosis, which may be reflected by high metabolic activity on FDG PET. Necrosis-targeting agents could be loaded and localized by injection into imaged regions of necrosis or high interstitial pressure, such as regions with restricted diffusion on MRI. Anti-vascular agents loaded on an image-able bead could be targeted to areas of high enhancement or vascular density, also easily localized on imaging. Fused imaging on top of procedural CBCT also allows exquisite mechanistic targeting, in such a paradigm^17^. Combining iodine-based and a bismuth-based beads loaded with different chemotherapy or immunotherapy drugs, could potentially enable dual drug delivery for a more rational, fusion image guided therapy grounded in mechanistic molecular reasoning. Such a paradigm could move image guided therapies into a new era after the field has delivered the same anthracycline for over four decades^18^.

The development of more advanced material decomposition-based algorithms, artificial intelligence-based CT image reconstruction, or artificial intelligence-based segmentation may further enable iodine/bismuth material classification and drug dose maps for treatment plans. Higher-resolution imaging and advanced material decomposition may be further explored in the next-generation CT scanners; photon counting CT, for example, can distinguish between bismuth, gadolinium, and iodine, in vivo^19^.

## Limitations and future work

Drug loading and elution profiles of the derived bismuth beads were not evaluated in this study. Based on the chemistry of the bismuth beads, it is expected that elution profiles will parallel its predecessor iodine-based radiopaque beads’ loading and elution profiles, but additional work will be required to confirm. The primary goal of the current study was to synthesize and characterize a radiopaque bismuth bead. In vitro evaluation of small agarose volumes containing beads may not reflect the full signal-to-noise levels observed in vivo with motion and in vivo environmental factors. In vivo preclinical studies were beyond the scope of this paper but are required to further explore the radiopacity and performance of the beads. However, the radiopacity of the beads on DECT was well above that of normal liver, so beads packed within hepatic arteries will be visible within the liver. While the bismuth is expected to remain bound to the chelation macrocycle^20,21^, in vivo safety studies are required, which may also include an assessment of the embolization or drug-eluting effect of the beads in tissues.

## Conclusion

In this study, we present facile methods for the synthesis of radiopaque polyvinyl alcohol-based microspheres, or beads, based on a marketed microsphere subsequently modified with a bifunctional macrocycle and chelated with bismuth. Bismuth distribution was uniform across the bead **6**. The radiopacity of the bismuth beads was confirmed by microCT and DECT. Furthermore, DECT imaging permitted material classification (decomposition) that could distinguish bismuth beads alone from iodinated contrast or a mixture of iodine and bismuth beads, which should also extend to discrimination between iodinated beads and bismuth beads. Imaging of two distinct bead populations may enable and promote the development of dual drug delivery and dual drug tracking when the drugs are delivered by beads that can be distinguished on imaging. The combination of drugs, devices, and imaging-guidance with dual drug mapping is enabled by dual bead localization, which could stimulate the field to evolve beyond a four decade history of using the same drug, doxorubicin, delivered via similar catheters with similar x-ray guidance.

## Materials and methods

### Materials

All reagents were purchased from commercial sources and used without further purifications. Acrylamido polyvinyl alcohol-*co*-acrylamido-2-methylpropane sulfonate microspheres, dried to a free-flowing powder and untainted, were obtained from Biocompatibles UK Ltd, (a BTG now Boston Scientific company, Farnham, UK). Bi(CF_3_SO_3_)_3_ was chosen as bismuth salt and was obtained from Sigma-Aldrich (Milwaukee, WI). SEM and EDX were used to evaluate surface smoothness and bismuth distribution across the bead, respectively (EMSL Analytical, Inc, Cinnaminson, NJ). Elemental composition was performed by Robertson Microlit Laboratories (Ledgewood, NJ). Material decomposition techniques were used to determine whether bismuth and iodine could be distinguished using DECT.

### Bismuth beads synthesis

#### General

Reaction progress was monitored by reverse-phase HPLC (Rp-HPLC) using a Beckman System Gold HPLC (Fullerton, CA) equipped with a 126 solvent module and 168 UV detector (λ = 254 nm) controlled by 32 Karat software and Beckman Ultrasphere C_18_ column (ODS, 4.6 × 250 mm, 5 μm), mobile phase: (A) 90% (0.1% TFA in water); (B) 10% (0.1% TFA in acetonitrile); 0–5 min, 10–90% B, 5–15 min, 90% B, 15–25 min. The flow rate was set to 1 mL/min. TLC was carried out on Merck silica gel 60 TLC plates F254 and visualized by UV at 254 nm. Column chromatography was performed using silica gel 60 (70–230 mesh). ^1^H and ^13^C were NMR recorded on a Bruker Avance 300 instrument with a deuterated solvent. Mass spectra were recorded with a Waters (Waltham, MA USA) LCT Premiere ESI TOF mass spectrometer. The instrument was operated in positive ion ESI mode at a mass resolution of 10,000.

### Synthesis of macrocycle

#### 4-Formylbenzyl-DO3A-tris-(*t*-Bu ester) (**2**)

The titled compound (**2**) was synthesized as described by Mondal et al.^22^ with slight modification. To a solution of DO3A-t-Bu-ester (**1**) (5 g, 9.7 mmol) and 4-(bromomethyl)benzaldehyde (1.93 g, 9.7 mmol) in anhydrous *N*, *N*-dimethylformamide (DMF) (60 mL) was added K_2_CO_3_ (1.6 g, 11.64 mmol). The reaction was stirred at room temperature for 4 h. The reaction progress was monitored by Rp-HPLC as described in the general method. The reaction mixture was filtered and the solvent was removed. The product was purified by column chromatography (silica gel, MeOH:DCM from 0–10%) to give (**2**) (4.7 g, 76%) dull yellow powder. ^1^HNMR (CDCl_3_, 300 MHz): δ ppm 1.5 (27H, s, ^t^Bu), 2.2–2.9 (16H, m, CH_2_), 3.0–3.1 (6H, brs, CH_2_COOtBu), 3.2 (2H, s, CH_2_), 7.7 (2H, d, J = 8 Hz, Ph), 7.9 (2H, d, J = 8 Hz, Ph), 10.0 (s, HCO); ^13^C NMR (CDCl_3_, 75 MHz): δ ppm 27.6, 49.7, 55.4, 55.7, 59.1, 82.2, 82.6, 129.7, 130.8, 135.3, 144.4, 172.4, 173.2, 191.5. MS–ESI: *m/z* (%) = 633.4 (100) [(M + H)^+^], HRMS[(M + H)^+^] = calcd. for: [C_34_H_57_N_4_O_7_]^+^ 663.4220; found 663.4227.

#### 4-Formylbenzoyl-DO3A-tris-(*t*-Bu ester) (**3**)

4-Formylbenzoyl-DO3A-tris-(*t*-Bu ester) (**3**) was made from 4-formylbenzoic acid (4-FBA) (20.91 g, 139.14 mmol). 4-Formylbenzoyl chloride was synthesized by the method of Iserloh et al*.*^23^. 4-Formylbenzoyl chloride was coupled with the DO3A-tris-(*t*-Bu ester) (60.01 g, 92.76 mmol) as described by Bhakuni et al. ^24^. The resulting reaction mixture was purified by column chromatography (Biotage Isolera One chromatography system on 340 g snap columns, Biotage Sweden) with a gradient solvent system of ethyl acetate:methanol; 100%:0% to 95%:5% to give the desired product, **3** as pale yellow viscous syrup (55.33 g, 73.4% yield and 97% HPLC purity); (^1^HNMR (CDCl_3_, 500 MHz): δ ppm 1.41 (9H, s), 1.45 (9H, s), 1.47 (9H, s), 2.70–2.68 (2H, m), 2.80–2.73 (6H, m), 2.91 (2H, *J* 6.5 Hz), 3.03 (2H, t, *J* 5.3 Hz), 3.07 (2H, s), 3.31 (2H, s), 3.32 (2H, s), 3.62 (2H, *J* 6.5 Hz), 3.79 (2H, t, *J* 5.3 Hz), 7.55 (2H, d, *J* 8.1 Hz), 7.90 (2H, d, *J* 8.1 Hz), 10.04 (1H, s); ^13^C NMR (CDCl_3_, 125 MHz): δ ppm; 28.16, 28.24, 44.67, 48.87, 52.00, 52.32, 52.82, 53.05, 53.52, 54.71, 54.98, 57.64, 58.16,, 80.89, 80.94, 80.98, 127.16, 129.81, 136.38, 143.32, 170.49, 170.64, 170.76, 170.80, 191.62.

#### Deprotection

##### DOTA-4-formylbenzyl (**4**)

Compound **2** (9.2 g, 14.6 mmol) was treated with 4 N HCl in dioxane (450 mL) by the method described by Muhlemann et al*.*^25^ to give DOTA-4-formylbenzyl (**4**). 8 g (qualitative yield) of dull white powder was obtained. MS–ESI: *m/z* (%) = 465.2 (100) [(M + H)^+^], HRMS[(M + 1)^+^]: calcd. for: [C_22_H_33_N_4_O_7_]^+^ 465.2345; found 465.2349.

##### DOTA-4-formylbenzoyl (**5**)

Compound **3** was treated using a similar method as described in compound **4** to give DOTA-4-formylbenzoyl (**5**) (yield, 87%, purity confirmed by HPLC).

#### Acetalation of the beads (**6**)

##### *Method I* Aromatic linker of compound **4**

*Method Ia* DMSO as a reaction solvent

1 g of beads (polyvinyl alcohol-co-acrylamido-2-methylpropane sulfonate precursor microspheres, Biocompatibles UK Ltd, (BTG/Boston Scientific), Farnham, UK) were swollen in 35 mL of anhydrous DMSO followed by adding 1 g of compound **4** (2.15 mmol). The resulting mixture was stirred for 30 min to distribute the macrocycle compound **4** into the swollen beads uniformly. Then, 2.2 mL of methanesulfonic acid was added dropwise from a syringe through a 30 G, 1/2-inch needle, and the reaction mixture was stirred at a lower stirrer setting under N_2_ at 50 °C for 26 h. The progress and the reaction rate were monitored with HPLC, every two hours for the first eight hours and then at 24 and 26 h, for the consumption of macrocyclic compound **4**. The reaction mixture was filtered. The filtrate was washed with DMSO (10 mL), then with copious amount of saturated sodium bicarbonate to completely remove the acid (monitored with litmus paper) and finally with deionized water. The acetalated beads (**6**) were used for the subsequent reaction.

*Method Ib* DMF as a reaction solvent

Acetalation of the beads was repeated as described in (a), except that the DMSO solvent was replaced with DMF solvent.

##### *Method II:* Aromatic linker of compound **5**

Acetalation of the beads with compound **5** was performed in accordance with the method of Duran et al*.*^10^ with the following adaptations: NMP (*N*-methyl pyrrolidinone) was the solvent used, and the reactions were performed at 55–65 °C for up to 40 h, monitored with HPLC for the consumption of the macrocycle. The liquid phase was removed, and the filtered beads were washed with NMP (90 mL), then suspended in water and neutralized with 0.1 M sodium carbonate to pH 8.5. The beads were then washed with water (90 mL) and NMP (250 mL) followed by neat NMP (150 mL). The acetalated beads (**7**) were used directly in the subsequent reaction.

#### Chelation of bismuth to the acetalated beads

Acetalation of the beads with compound **4** was performed with two different reaction solvents: dimethyl sulfoxide (DMSO) and dimethylformamide (DMF).

##### Method I bead (**6**)

The acetalated beads (**6**) (Synthetic Scheme 1) were resuspended in deionized water at a pH of 8–9 using 0.1 N of sodium bicarbonate, and Bi(CF_3_SO_3_)_3_ (4 mmol) was added and stirred at 80 °C for 1 h. The final beads were filtered and washed with an aqueous solution of sodium bicarbonate (0.1 M) and finally with copious amounts of deionized water to remove residual bicarbonate (monitored by litmus paper). The complete removal of bismuth salt was confirmed using an aqueous solution of potassium iodide (1%, w/v) as reported in the literature^26^.

##### Method II beads (**7**)

Acetalated beads (**7**) were suspended in anhydrous NMP (30 mL) under N_2_. Pyridine (0.54 mL) was added, then the mixture was warmed to 55 °C, followed by addition of bismuth trifluromethanesulfonate (2.455 g, 1.5 eq.). The resulting suspension was stirred overnight at 55 °C under N_2_. The liquid portion was removed and the filtered beads were washed with NMP (250 mL). The beads were then suspended in phosphate buffered saline, neutralized to pH 7 with 0.1 M Na_2_CO_3_ and then washed with phosphate buffered saline (250 mL).

#### Sterilization

Bulk beads were dispensed into 20 mL Schott FIOLAX clear glass vials and sealed with bromobutyl Fluro Tec injection stoppers and FOTO caps and autoclaved at 121 °C for 30 min as reported previously^10^.

### Bismuth beads characterization

#### Bead physical property

The solid content of the hydrated bead, bismuth concentration of hydrated bead, and the bismuth content of the dry beads of **7** were determined as described previously^10^ (see SI). Both dry bismuth beads (**6** and **7**) were analyzed for their elemental composition by Robertson Microlit Laboratories (Ledgewood, NJ).

#### Microscopic evaluation

Beads (**7**) were sieved into four sizes ranging between 100–600 mm in diameter and evaluated under light microscopy for surface deformation and purity after the reaction process. The size and appearance of beads were examined^9^. Briefly, an aqueous suspension of 150 ml of beads in deionized water was placed on a chamber slide (Electron Microscopy Sciences). Bright-field images were acquired with a 5 × objective on an upright microscope (Zeiss Axioimager M1, Thornwood, NY) equipped with a color CCD camera (AxioVision, Zeiss).

#### Bismuth mapping across the beads using SEM–EDX

The beads were embedded in various mounting media, including Norland Optical Adhesive, Epoxy Resin, Polyester Resin, and Paraffin. The Norland Optical Adhesive media was used for the preparation due to its sulfur content, which aids in determining the particle edge during mapping and line scan analysis. The preparations were performed on aluminum stubs used during scanning electron microscopy. Each mount was sectioned using a microtome equipped with liquid nitrogen and glass knives. After sectioning, the preparations were evaluated for sample integrity and the prepared sections coated with ~ 300 angstroms of gold. The visualization of the synthesized bismuth beads was carried out in a JOEL Scanning Electron Microscope, model JEM-6510 equipped with tungsten filament. Elemental analysis for bismuth was determined using EDX spectroscopy (Oxford) by using 20 keV energy, and the line scans were generated using Aztec software. Multiple line scans through the microsphere cross-sections of the bismuth elemental map were acquired for each bead population to show the distribution of bismuth from edge to edge.

#### Catheter delivery performance

Catheter deliverability of the bismuth beads through two clinical microcatheter sizes (2.0-Fr Progreat, Terumo, Somerset, NJ and 2.4 Fr Renegade, Boston Scientific Corp.) was evaluated by administering a homogenous bead suspension in 100% iohexol (Omnipaque 350, GE Healthcare, Waukesha, WI) as a 1:10 bead dilution and using 1- or 3-mL syringes^10^. The catheters were laid on a bench with a 10 cm diameter coil mid-catheter to introduce curvature in the flow path. Any catheter clogging was recorded as a failure and denoted that bead size was not suitable to the respective catheter.

### Bismuth Beads Imaging

The bead with the higher bismuth content and bismuth homogeneity across the bead surface was chosen for microCT and DECT evaluation.

#### Imaging phantom preparation

A suspension of bead in agarose gel (1 mL, 0.5% total agarose) was used to assess the radiopacity on microCT^9^. To assess radiopacity and material decomposition with DECT, iodine, bismuth beads, or a mixture of both were added to 1% agarose for a total volume of 1 mL For the iodine-agarose gels, four tubes were prepared using the clinical contrast agent Isovue-300 with 5, 10, 20, and 40 mg I/mL per tube. For the bismuth-agarose tubes, four tubes were prepared using suspensions of bismuth beads **6**, with 62.5, 125, 250, and 500µL bismuth beads/mL per tube. Similarly, agarose tubes with mixtures of Isovue-300 and bismuth beads were prepared with the following ratios of mg iodine/mL and µL bismuthbeads/mL: 10:125, 10:250, and 20:125.

#### MicroCT

MicroCT was performed to evaluate the radiopacity of individual bismuth beads suspended in agarose ^9^. Homogeneously dispersed beads in an agarose phantom (1 mL, 0.5% total agarose) were scanned with Bruker SkyScan1272 (Kontich, Belgium) microCT at a nominal resolution (pixel size) of 5.0 microns with a 0.5 mm thick aluminum filter, a source current 125 mA, and an applied X-ray tube voltage of 80 kV. Reconstruction was carried out with a modified Feldkamp algorithm using the SkyScan NRecon software (Kontich, Belgium). Ring artifact reduction and beam hardening correction were applied. Image post-processing was performed (CT Analyser, version 1.20.3.0, Kontich, Belgium). Beads within the 70 and 150 μm range were selected for post-analysis, while the beads in or near a saturated spot were excluded. The voxel values were converted to HU using a water calibration phantom. A 3D mask was created for each bead to enable the calculation of HU per bead and analyze the variation in mean density per bead size.

#### Dual energy CT

DECT imaging of the agarose gels was performed on a 192-slice DECT scanner (SOMATOM Force Dual Source, Siemens Healthcare GmbH, Forchheim, Germany). Clinical scanning protocols for the abdomen/pelvis were used with 80 kVp imaging paired with Sn150 kVp and 100 kVp paired with Sn150 kVp, where Sn150 kVp refers to the use of tin filtration of the X-ray beam. Images were reconstructed at 0.5 mm slice thickness. The tubes were inserted in a test tube rack (Supplementary Fig. 2), immersed in a water-filled container, and imaged in triplicate. Image post-processing of the data was performed with Matlab (MATLAB 2017b and Statistics Toolbox and Image Processing Toolbox, The MathWorks, Inc., Natick, MA, US).

##### Dual Energy Index (DEI)

Linear regression was performed between measured CT numbers versus the known iodine concentration or bismuth bead volume for both pairs of imaging exposures. The dual-energy index (DEI) was calculated from the CT numbers using the low and high energy images of each pair as follows^27,28^.

$$DEI_{i} = (HU_{i}^{low} - HU_{i}^{high} )/(HU_{{i{ }}}^{low} + HU_{i}^{high} + 2000)$$

where *HU*_*i*_^*low*^ and *HU*_*i*_^*high*^ are the HU values of material *i* at the low energy acquisition (80 or 100 kVp) and the high energy acquisition (Sn150 kVp), respectively. The individual attenuations are shifted by 1000 HU to avoid negative values, which results in the factor of 2000 HU in the denominator in the reduced equation^29^. The acquisition pair that maximizes the difference in HU for iodine, while minimizing the difference in HU for bismuth was chosen for further evaluations.

In addition, the threshold value of DEI that maximally separates the bismuth and iodine was determined, using an optimizer function (R Vienna, Austria. URL https://www.R-project.org/). A DEI image was created, and values above the threshold value were displayed as a red color map superimposed on the original DEI image. A combined map of DEI and HU values above their respective thresholds was created by blending the two maps and then superimposing the results onto the low kVp acquisition.

## Supplementary information

Supplementary Information.
