# Supplementary material for: Synthesis, characterization, and imaging of radiopaque bismuth beads for image-guided transarterial embolization
Source: Sci Rep. 2021 Jan 12;11:533. doi: 10.1038/s41598-020-79900-z (PMC7804415; doi:10.1038/s41598-020-79900-z)
Supplement: Supplementary file 1 — Supplementary Information. [file 41598_2020_79900_MOESM1_ESM.docx]

**Synthesis, characterization and imaging of radiopaque bismuth beads for image-guided transarterial embolization**

***Ayele H. Negussie^1,5^, Quirina M.B.*** *de Ruiter^1^, Hugh Britton^2^, Danielle R Donahue^3^ , Quentin Boffi^2^, Young-Seung Kim^3^, William F. Pritchard^1^, Chrit Moonen^4^, Gert Storm^5,6^, Andrew L. Lewis^,^  Bradford J. Wood^1^*

**Determination of solid content and bismuth concentration of hydrated beads**

To calculate the equilibrium water content and solid content, between 0.2 and 0.3 mL of sedimented beads (bead **7**) were washed 3 times with 10 mL of deionized water and placed on a shaker plate at 250 rpm for 5 minutes in order to remove residual salts present in the packing solution. The washed beads were then transferred to a pre-weighed aluminum plate and a minimum amount of deionized water to aid the complete transfer of beads. Excess water was removed using a Pasteur pipette followed by pressing a filter paper gently against the beads. The aluminum plates containing beads were then transferred to a vacuum oven and the samples were allowed to dry at a pressure of 50-100 mbar and 50 °C. After 12 hours, the aluminum plates containing the dried beads were weighed to calculate equilibrium water content.

**Catheter Delivery Performance**

Catheter deliverability of the bismuth beads through two clinical microcatheter sizes (2.0-Fr Progreat, Terumo, Somerset, NJ and 2.4 Fr Renegade, Boston Scientific Corp.) was evaluated by administering a homogenous bead suspension in 100% iohexol (Omnipaque 350, GE Healthcare, Waukesha, WI) as a 1:10 bead dilution and using 1- or 3-mL syringes, mixed with Omnipaque 350, through two clinical microcatheter sizes (2.0F and 2.4F) was evaluated ^10^. The deliverability of the beads, mixed with iohexol contrast (Omnipaque 350, GE Healthcare, Waukesha, WI ), through two clinical microcatheter sizes (2.0F and 2.4F) was evaluated as reported previously 10. Catheter deliverability was determined by administering a homogenous bead suspension in 100% iohexol (1:10 bead dilution, 1- or 3-mL syringe). The catheters were laid on a bench with a 10 cm diameter coil mid-, and at the center of the catheter a coil (10 cm diameter) was introduced to produce a more tortuous to introduce curvature in the flow path for the beads to navigate thorough (a curvature catheters may experience in a patient). Any catheter clogging was recorded as a failure and denoted that bead size was not suitable to the respective catheter. Catheters included in this study are 2.0-Fr Progreat (Terumo, Somerset, NJ) and 2.4 Fr Renegade (Boston Scientific Corp.).


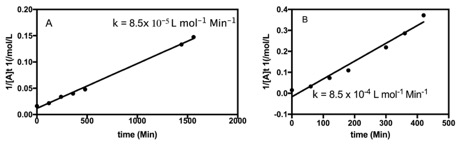


**Supplementary Figure 1.** Acetalation reaction of the beads with **4** in A) DMSO and B) in DMF with second-order reaction rate with rate constant k = 8.5× 10^-5^ L mol^-1^Min^-1^ and k = 8.5× 10^-4^ L mol^-1^Min^-1^, respectively. Where [A]t is concentration of 4-(bromomethyl)benzaldehyde over time.

**Supplementary** **Table 1**: Physical characterization of different sizes of bismuth beads (radiopaque bead 7)

| **Properties** | **Bismuth bead size (mm)** | | | |
| --- | --- | --- | --- | --- |
|  | **100-160** | **250-355** | **355-425** | **425-600** |
| **Physical characterization** | | | | |
| **Solid content of hydrated bead (mg/mL)** | 195.4 | 190.9 | 157.1 | 169.7 |
| **Bismuth concentration (mg/mL) hydrated bead** | 69.3 | 49.5 | 48.6 | 51.2 |
| **Delivery performance** | | | | |
| **Deliverability (2.0 Fr) – (hydrated beads diluted in contrast)** | Yes | Yes-moderate | No | No |
| **Deliverability (2.4 Fr) (hydrated beads diluted in contrast)** | Yes | Yes | Yes-moderate | No |

**Supplementary** **Table 2**: Mean and SD of the Hounds field voxel values for each tube in 80,150kv and the DEI.

| **Tube** | **Iodine**  **Component** | **Bismuth**  **Component** | **80kV HU**  **mean(SD)** | **150kV HU mean(SD)** | **DEI**  **mean(SD)** | **DEI > 0.06 (%)** |
| --- | --- | --- | --- | --- | --- | --- |
| **Iodine Contrast (mgI/mL)** | | | | | | |
| 5 | 12.50% | 0% | 246 (37) | 73 (40) | 0.07 (0.02) | 74.3% |
| 10 | 25% | 0% | 442 (34) | 124 (38) | 0.12 (0.02) | 99.9% |
| 20 | 50% | 0% | 901 (40) | 258(40) | 0.20 (0.02) | 100.0% |
| 40 | 100% | 0% | 1660 (65) | 490(43) | 0.28 (0.02) | 100.0% |
| **Bismuh beads (mL beads/mL)** | | | | | | |
| 62.5 | 0% | 12.50% | 103 (45) | 84(40) | 0.01 (0.02) | 0.3% |
| 125 | 0% | 25% | 235 (72) | 184 (65) | 0.02 (0.02) | 3.4% |
| 250 | 0% | 50% | 496 (166) | 395(140) | 0.03 (0.02) | 9.6 % |
| 500 | 0% | 100% | 916 (196) | 736(169) | 0.05 (0.02) | 25.8% |
| **Mixed (mL beads /mL + (mL beads/mL)** | | | | | | |
| 125 Bi+10 I | 25% | 25% | 690(119) | 343 (109) | 0.11 (0.02) | 99.7% |
| 125 Bi+20 I | 50% | 25% | 1111(77) | 452 (69) | 0.19 (0.02) | 100.0% |
| 250 Bi + 20I | 50% | 50% | 1279 (74) | 620 (66) | 0.17 (0.02) | 100.0% |

A


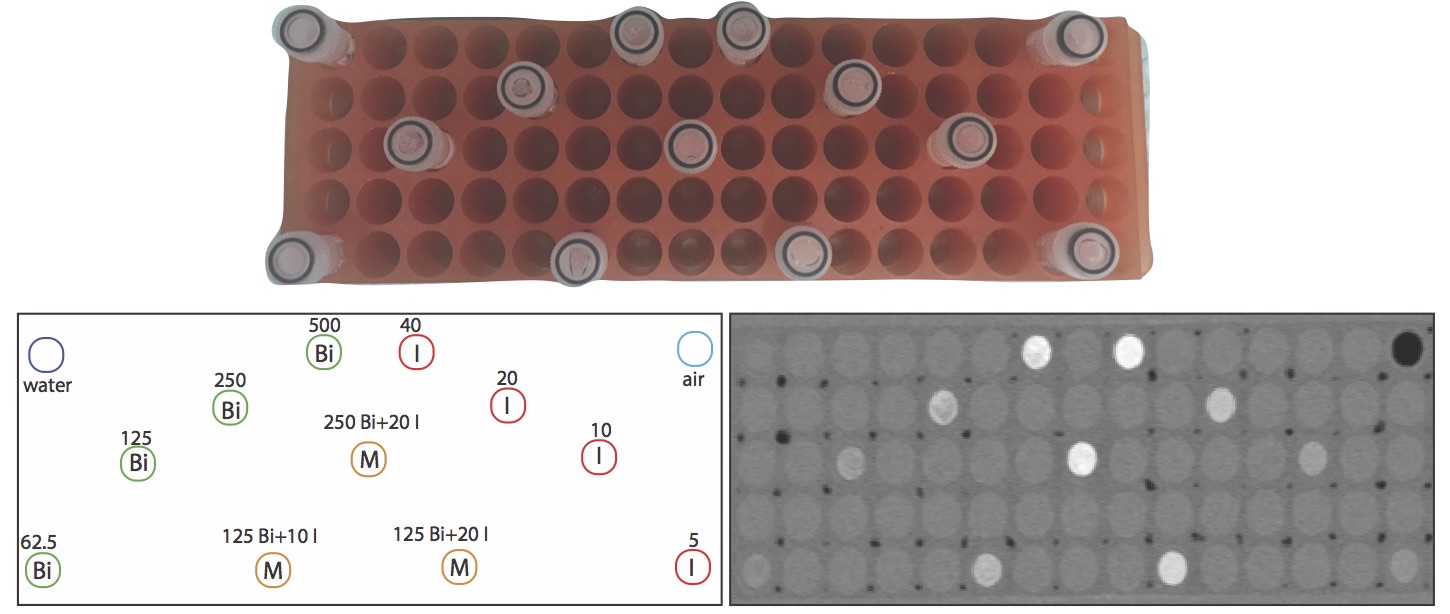


C

B

**Supplementary Figure 2.** DECT of agarose tubes with iodine contrast (mg I/mL, red circles), bismuth beads (mL beads/mL, green circles) or a mixture (orange circle). A) Arrangement of tubes in the rack. B) Tube contents and concentrations in agarose. C) Axial CT acquisition from the Sn150 kVp scan.
